# First report of *Mesocriconema xenoplax* (Nematoda: Criconematidae) from turfgrass in Portugal and in Europe

**DOI:** 10.21307/jofnem-2019-035

**Published:** 2019-07-23

**Authors:** M. L. Inácio, L. C. Rusinque, M. J. Camacho, F. Nóbrega

**Affiliations:** 1 Instituto Nacional de Investigação Agrária e Veterinária (INIAV, I.P), Quinta do Marquês, 2780-159, Oeiras, Portugal

**Keywords:** Detection, Lawn disease, Ring nematode, Tall fescue

## Abstract

In Winter 2016, root and soil samples were collected from the rhizosphere soil at 10 to 15 cm depth of turfgrass, in the yard of a complex of houses in Caxias, a region near Lisbon, Portugal. The grass (dominated by tall fescue) showed yellow patches, stunting, and poor growth. Several specimens of a ring nematode (50-60 nematodes/100 ml soil) were recovered from soil and identified as *Mesocriconema xenoplax* (peach ring nematode) based on morphological and morphometrical analysis of females. The observed morphological features were with previous descriptions. Species identification was confirmed through sequencing of the fragment spanning D2/D3 domain of the 28S rDNA gene. Phylogenetic analysis revealed that the Portuguese isolate grouped with *M. xenoplax* isolates (98% similarity), supporting its identification as *M. xenoplax*. This is the first report of *M. xenoplax* from turfgrass in Portugal and in Europe contributing with additional information on the distribution of this phytoparasite.

Turfgrasses are among the most widely used ornamental plants in the world, serving important functions in soil stabilization and providing safe surfaces for recreational activities (Zeng et al., 2012a). Moreover, the quality of the turf in the sports areas, mainly football fields and golf courses, is crucial and any imperfection can have a huge impact (Oliveira et al., 2018). Of all turfgrass pests, nematodes are probably the least understood and most often overlooked. Due to this, nematode symptoms are often misdiagnosed because they appear similar to other factors such as localized soil conditions, fungal diseases, or insect attack. Ring nematodes can cause significant damage to grass if population densities are high enough. Feeding results in tiny lesions on the roots, and under high nematode pressure, roots can become discolored and stubby (Crow, 2005). Nematode damage usually appears as irregularly shaped declining areas in the lawn that may enlarge slowly over time. Grass will die under extreme nematode and environmental stress and often, as the grass thins out, spurge and other weeds may become prominent.

The plant parasitic nematode *Mesocriconema xenoplax* (=*Criconemoides xenoplax*, Raski, 1952) Loof and De Grisse, 1989 is a root damaging ectoparasite with a worldwide distribution and a wide host range, comprising grapevine, all *Prunus* species, walnut, lettuce, carnation, pine, and grasses. Its presence has been reported in vineyards in several countries around the world and is a major factor in peach tree short life (PTSL), a syndrome that results in premature mortality of peach trees in the southeastern USA (Nyczepir et al., 1983; Nyczepir, 2011). It also was found in walnut in Italy (Ciancio and Gaetano, 1998) and around the USA in turfgrass (Zeng et al., 2012b) among others. In Portugal, its presence was reported for the first time in fig trees in 2008 (Abrantes et al., 2008), but no further reports of its presence have been made.

## Materials and methods

**Figure 1:**
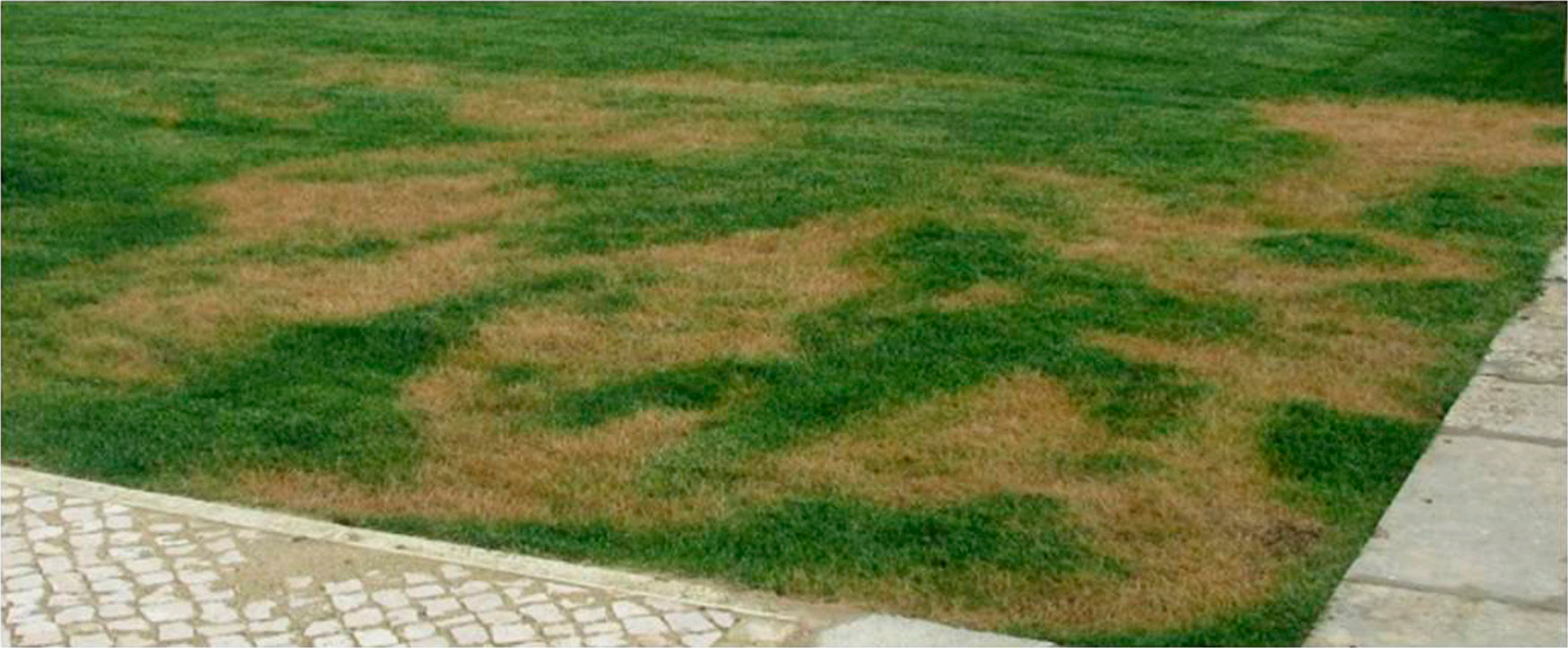
Symptoms of *Mesocriconema xenoplax* infestation in turfgrass.

In Winter 2016, yellow and declining patches were observed in the yards of a complex of houses in Caxias (Lisbon district), Portugal (Fig. 1). The instant roll-out lawn turf in the yard was a mixture provided by a private lawn and landscape company, with tall fescue as the predominant species (70% *Schedonorus arundinaceus* (Schreb.) Dumont. formerly *Festuca arundinacea* Schreb., 15% perennial ryegrass, *Lolium perenne* L., and 15% bluegrass, *Poa pratensis* L.).

To identify the problem, soil samples were collected after digging up to the depth of 10 to 15 cm along the margins of the chlorotic areas. Each sample consisted of 5 to 8 cores (30 mm diameter) sampled at roughly equal intervals following the patches across an area of 1000 m^2^ or less. Six composite soil samples were placed in polyethylene bags and immediately brought to laboratory for analysis. A 500 mL subsample was taken from each composite sample and processed to identify and count plant parasitic nematodes. Nematodes were extracted following the sieving and decanting technique (EPPO, 2013). The nematodes were collected from all samples and identified to genus level. At least 10 were placed in a drop of water on a glass slide and gently heat killed for morphological characterization using a brightfield light microscope (Olympus BX-51, Hamburg, Germany) and photographed with a digital camera (Olympus DP, Hamburg, Germany).

To confirm the morphological identification, DNA from selected female specimens was used for sequencing of the D2/D3 expansion segments of the 28S ribosomal RNA, following Subbotin et al. (2005). In total, 10 nematodes (juveniles and females) were handpicked and transferred individually to Eppendorf tubes with 10 µl of sterilized water, for DNA extraction, PCR amplification, and sequencing. Each nematode was frozen in liquid nitrogen and homogenized with a micro-pestle (Eppendorf, Hamburg, Germany). The homogenate was incubated at 56°C in lysis buffer and 100μg ml-1 proteinase K for 1 hr. After incubation, total genomic DNA extraction was performed using the DNeasy Blood & Tissue Kit (Qiagen, Hilden, Germany) following the manufacturer’s instructions. DNA quantity and purity were checked using a NanoDrop 2000 UV-V is Spectrophotometer (Thermo Fisher Scientific, Massachusetts, USA). PCR mixtures, using the primers D2A (5′-ACAAGTACCGTGGGGAAAGTTG-3′) and D3B (5′-TCGGAAGGAACCAGCTACTA-3′) (De Ley et al., 1999) and thermal cycling conditions were performed as described previously by Inácio et al. (2016). PCR products were cleaned using the QIAquick PCR Purification Kit (Qiagen, Hilden-Germany), according to the manufacturer’s instructions. Amplicons were sequenced at STABVida Sequencing Laboratory (Lisbon, Portugal) on a DNA analyzer ABI PRISM 3730xl (Applied Biosystems). Nucleotide sequences were edited and analyzed using BioEdit v7.2.0 (Hall, 2007). Sequencing of the one-single nematode PCR products resulted in sequences totally identical thus only one was included in this study. The resulting D2/D3 rDNA sequence was compared against a set of reference sequences of *M. xenoplax* selected from GenBank (NCBI) to cover a range of species from Criconematidae. Nucleotide sequences from isolates for which GenBank sequence data were available for the homologous fragment of D2/D3 rDNA gene were retrieved and a new multiple alignment was performed, using ClustalW integrated in software MEGA 6 (Tamura et al., 2013).

A phylogenetic tree was estimated under maximum likelihood (ML) based on the model that best fit the data, which was identified as a Tamura 3-parameter model also integrated in MEGA 6. In total, 1,000 bootstrap replicates were performed to test the support of each node on the trees. One species of the genus *Hemicriconemoides*, *H. promissus*, was used in the phylogenetic analyses as outgroup. Sequences selected from the GenBank database and the access numbers were plotted in the phylogenetic tree. Estimates of evolutionary divergence between sequences were calculated using a Kimura 2-parameter distance model.

## Results and discussion

From the recovered nematodes (50-60 nematodes/100 ml soil), morphological characterization showed the affinity of specimens with *M. xenoplax* from previous descriptions. The female exhibited a long, wide stylet, with anchor-shaped knobs. The head was broad, and with an elevated labial disc, and the first cephalic annulus was indented or anteriorly projected. The tail was broadly round, and the terminus was most often a small and rounded button. The vulva was distinctly open. Juveniles were much smaller than the adults, resembling adult females. The morphology (Fig. 12A,B) and measurements of 10 females were compared to the descriptions of *M. xenoplax* made by Loof and De Grisse (1989) (Table 1) and subsequent studies (Cordero et al., 2012), and measurements were consistent with the results of these studies. No other active plant parasitic nematodes were found in suspensions extracted from the soil samples.

**Table 1.** Comparison of the gross range of morphometrics recorded of the Portuguese population of *Mesocriconema xenoplax* (all measurements are in µm and in the format mean ± SD (range)), with the descriptions of Loof and De Grisse (1989).

Character/ratio	Gross range (10♀♀)	As per Loof and De Grisse (1989)
L	685 ± 20.7 (650–700)	590 (560–670)
a	13.9 ± 0.9 (12.0–14.6)	13.2 (12.0–15.0)
b	4.2 ± 0.1 (4.1–4.3)	4.2 (4.0–4.3)
c	20.8 ± 1.3 (19.4–23.3)	21.0 (19.0–23.4)
%V	92 ± 0.9 (91–93%)	92 (91–93%)
Stylet	77.5 ± 2.1 (75.0–81.0)	81.0 (77.5–85.0)
R	113 ± 1.9 (108–114)	109 (104–116)
Ran	5–8	7–8
RVan	0–4	–
RV	7–8	8–9

Notes: R, total number of body annules; Ran, number of body annules between anus and tail tip; Rvan, number of body annules between vulva and anus; RV, number of body annules between vulva and tail tip.

**Figure 2:**
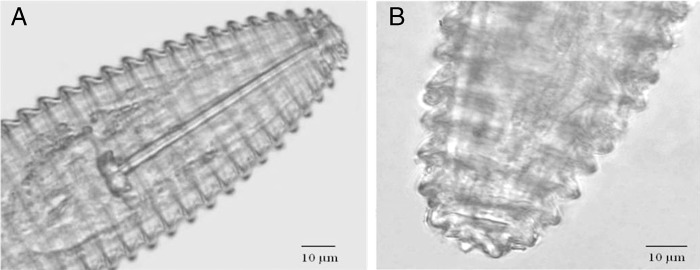
Light micrographs of *Mesocriconema xenoplax* (female). (A) Anterior body portion showing lip region and the long stylet; (B) posterior body portion showing rounded tail shape.

One of the nucleotide sequences obtained in this study was deposited into the GenBank database (NCBI) under the accession number MG647831. Amplification of the D2/D3 rDNA loci resulted in a PCR product of 552 bp and the nucleotide sequence showed a similarity range between 88 and 98% with isolates of *M. xenoplax* from different regions of the world.

**Figure 3:**
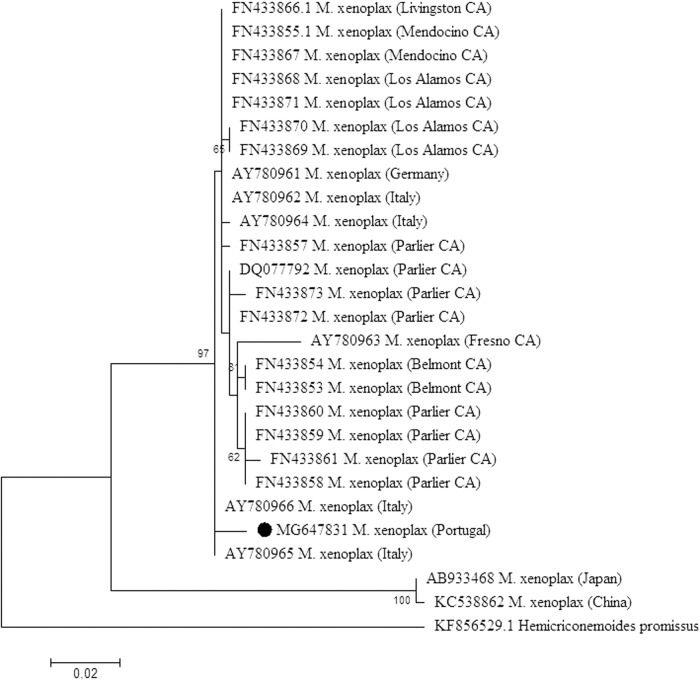
Phylogenetic relationships of *Mesocriconema xenoplax* collected from Portugal (MG647831) and other geographical regions, based on the sequence alignment of the D2/D3 rDNA loci. The phylogram was generated using the maximum likelihood method based on the Tamura 3-parameter model with 1,000 bootstrap replications. Bootstrap values are indicated at the nodes. The analysis involved 27 nucleotide sequences. All positions containing gaps and missing data were eliminated. Evolutionary analyses were conducted in MEGA 6.

The molecular phylogenetic status of samples as inferred from their D2/D3 sequences is presented in Figure 3. The phylogram reveals one clade, supported by a bootstrap value of 100%, that includes only two isolates of *M. xenoplax*, one from China (KC538862) and another from Japan (AB933468). All other isolates formed a separate major clade with a bootstrap value of 97%. The sequence divergence values ranged between 0 and 11% within all isolates. The isolates used for comparison presented values ranging from 0 to 2%, except those from China and Japan.

The identification of *M. xenoplax* from turfgrass (tall fescue + perennial ryegrass + bluegrass) constitutes the first report of this species from these plants in Portugal and in Europe. The nematodes were recovered in low numbers, most likely due to the cool season. On the other hand, it could be inferred that the symptoms exhibited did not correspond to abiotic damages since the turfgrass mixture used can resist low temperatures and was well managed. The yellow patches of plants caused by ring nematode infestation are undesirable symptoms that have a negative effect on lawns. In view of the touristic impact of the regions surrounding Lisbon that rely on extensive areas of gardens, parks, and golf courses, it is of great concern and of economic and environmental responsibility to avoid the spread of these parasitic nematodes. Therefore, good practices for managing their populations and to prevent loss of amenity value of the affected lawns are of primary importance among which, proper irrigation and fertilization and avoidance of other stresses on the grass are the most relevant. Moreover, using certified sod and planting material free of parasitic nematodes and performing soil analyses before lawn installation in gardens and golf courses is advised to avoid *M. xenoplax* geographical expansion in Portugal and elsewhere in Europe.
